# Incidence, mechanism and prognostic value of activated AKT in pancreas cancer

**DOI:** 10.1038/sj.bjc.6601396

**Published:** 2003-11-25

**Authors:** M G Schlieman, B N Fahy, R Ramsamooj, L Beckett, R J Bold

**Affiliations:** 1 Department of Surgery, University of California Davis Medical Center, Sacramento, CA 95817, USA; 2 Department of Pathology, University of California Davis Medical Center, Sacramento, CA 95817, USA; 3 Department of Epidemiology and Preventative Medicine, University of California Davis Medical Center, Sacramento, CA 95817, USA

**Keywords:** pancreatic cancer, AKT, HER-2/*neu*

## Abstract

When activated, the serine/threonine kinase AKT mediates an antiapoptotic signal implicated in chemoresistance of various cancers. The mechanism(s) of AKT activation are unknown, though overexpression of HER-2/*neu* has been implicated in breast cancer. Therefore, we determined the incidence of activated AKT in human pancreatic cancer, whether HER-2/*neu* is involved in AKT activation, and if AKT activation is associated with biologic behaviour. HER-2/*neu* expression and AKT activation were examined in seven pancreatic cancer cell lines by Western blotting. The *in vitro* effect of HER-2/*neu* inhibition on AKT activation was similarly determined. Finally, 78 pancreatic cancer specimens were examined for AKT activation and HER-2/*neu* overexpression, and correlated with the clinical prognostic variable of histologic grade. HER-2/*neu* was overexpressed in two of seven cell lines; these two cell lines demonstrated the highest level of AKT activation. Inhibition of HER-2/*neu* reduced AKT activation *in vitro*. AKT was activated in 46 out of 78 (59%) of the pancreatic cancers; HER-2/*neu* overexpression correlated with AKT activation (*P*=0.015). Furthermore, AKT activation was correlated with higher histologic tumour grade (*P*=0.047). Thus, it is concluded that AKT is frequently activated in pancreatic cancer; this antiapoptotic signal may be mediated by HER-2/*neu* overexpression. AKT activation is associated with tumour grade, an important prognostic factor.

It is well established that part of the phenotype of cancer cells is resistance to apoptosis, though the cellular mechanisms that mediate this characteristic are not fully understood. AKT has been recently demonstrated to be a major mediator of survival signals in a variety of cells, including cancer cells (Tang *et al*, 2001; Vivanco and Sawyers, 2002). Constitutive activation of AKT provides a potent antiapoptotic signal in cancer cells conferring chemo- and radioresistance (Brognard *et al*, 2001; West *et al*, 2002). To date, there have been a few studies on the incidence of AKT activation in various tumours using archived pathologic specimens (Dhawan *et al*, 2002; Gupta *et al*, 2002; Itoh *et al*, 2002; Malik *et al*, 2002; Perez-Tenorio and Stal, 2002). While these reports correlate AKT activation with some biologic property of the tumour, none has examined potential upstream activating mechanisms. In prostate cancer, the AKT activation occurred in 33 out of 125 specimens and was more frequent in higher Gleason grade cancers (Malik *et al*, 2002); in colon cancer, the AKT activation was observed in 30 out of 65 tumours and associated with advanced clinicopathologic stage (Itoh *et al*, 2002); in breast cancer, the AKT activation was frequently observed (50 out of 93) and predicted a worse prognosis in endocrine treated patients (Perez-Tenorio and Stal, 2002); in melanoma, the AKT activation was observed in eight out of 12 specimens and the incidence of AKT activation was more common than in precursor lesions (Dhawan *et al*, 2002); and in head and neck squamous cell carcinoma, the AKT activation was observed in 25 out of 38 (66%) specimens and correlated with local recurrence (Gupta *et al*, 2002).

Pancreatic cancer is a highly lethal malignancy resistant to the apoptosis-inducing effects of radio- and chemotherapy (Bardeesy and DePinho, 2002; Heinemann, 2002). Recent reports have suggested that AKT, a target of phosphatidylinositol 3-kinase (PI3K), is phosphorylated and thus activated under basal conditions in a variety of pancreatic cancer cell lines, possibly contributing to the inherent apoptotic-resistance (Xue *et al*, 2000; Bondar *et al*, 2002; Samatar *et al*, 2002). These data come from the available cell lines, though no data are available on the incidence of AKT activation in human tumours. Recent reports have demonstrated that the AKT pathway is a potent survival signal in these pancreatic cancer cell lines, as inhibition of PI3K, the upstream activator of AKT, has been shown to sensitise these cells to the apoptotic effect of chemotherapy *in vitro* (Ng *et al*, 2000; Perugini *et al*, 2000; Yao *et al*, 2002). Furthermore, using these cell lines in animal studies, inhibition of PI3K was well tolerated and increased the efficacy of chemotherapy *in vivo* (Ng *et al*, 2000; Bondar *et al*, 2002). Whether AKT represents an important survival signal and is an appropriate target for molecular-based therapy awaits validation of the incidence and clinical consequence of AKT activation in pancreatic cancer specimens.

There has been intense investigation into the potential upstream events that mediate activation of AKT in cancer cells. Various mediators that have been identified are PTEN (Haas-Kogan *et al*, 1998), K-ras (Guerrero *et al*, 2000), and HER-2/*neu* (Zhou *et al*, 2001), though other receptor tyrosine kinases have also been shown to be involved in AKT activation. *In vitro* analysis shows tumour specific differences, as PTEN is likely the dominant mechanism in glioblastoma multiforme (Sano *et al*, 1999) and prostate cancer (Davies *et al*, 2002); in breast cancer, HER-2/*neu* is likely the upstream activator (Zhou *et al*, 2000). In pancreatic cancer, Matsumoto *et al* (2002) recently demonstrated that neither mutation of K-ras or PTEN is associated with activation of AKT, though HER-2*/neu* signalling was not investigated. Despite these *in vitro* studies, there is little information on which signalling pathway is present in human tumours.

Therefore, we sought to determine whether AKT is activated in the tumours of patients with pancreatic cancer, whether this is associated with HER-2/*neu* overexpression, and the biologic consequence of these events.

## MATERIALS AND METHODS

### Materials

Cell culture supplies and media were purchased from Becton Dickinson (San Diego, CA, USA) and Gibco/BRL Life Technologies (Gaithersburg, MD, USA), respectively. Mouse monoclonal antibody to HER-2/*neu* for Western blotting was obtained from Oncogene Research Products (San Diego, CA, USA), while the mouse monoclonal antibody to HER-2/*neu* for immunohistochemistry was obtained from Dako Corp. (Carpinteria, CA, USA). Detection of the cellular level of AKT as well as the activated, phosphorylated form of AKT (phospho-serine-473) was through the use of polyclonal antibodies (Cell Signaling Technology, Beverly, MA, USA). Inhibition of HER-2/*neu* was performed using trastuzamab (Herceptin®, Genentech, South San Francisco, CA, USA).

### Cell culture

The seven human pancreatic adenocarcinoma cell lines were obtained from the American Type Culture Collection (Rockville, MD, USA). Cells were cultured in appropriate culture medium supplemented with 10% fetal calf serum, sodium pyruvate, nonessential amino acids, L-glutamine, penicillin/streptomycin antibiotics. Cells were maintained in a humidified incubator containing 10% CO_2_ at 37°C. Cells underwent serum starvation for the 24 h prior to treatment with trastuzamab.

### Western blotting

Following treatment, cells were harvested and lysed in a buffer containing 150 mM NaCl, 1% Triton X-100 and 25 mM Tris (pH 7.5). Debris was sedimented by centrifugation for 5 min at 12 000 **g**, and the supernatants were solubilised for 5 min at 100°C in Laemmli's sodium dodecyl sulphate–polyacrylamide gel electrophoresis (SDS–PAGE) sample buffer containing 100 mM dithiothreitol. Protein concentrations of the lysates were determined with a protein quantitation kit (Bio-Rad Laboratories, Hercules, CA, USA), and 50 *μ*g of each sample was separated on a 10% SDS–PAGE gel. Separated polypeptides were then electrophoretically transferred to 0.2-mm nitrocellulose membranes (Schleicher & Schuell, Keene, NH, USA). Membranes were blocked for 1 h in a Tris-buffered saline-Tween (TBS-T; 25 mM Tris, pH 8.0, 150 mM NaCl, and 0.05% Tween-20) containing 5% (w v^−1^) nonfat dried milk. Blots were then probed overnight with primary antibodies and developed using species-specific secondary antisera. Immunoreactive material was detected by the enhanced chemiluminescence technique (Amersham, Piscataway, NJ). Relative polypeptide expression was quantified by laser densitometry (Molecular Dynamics, Sunnyvale, CA, USA).

### Identification of pancreatic adenocarcinoma specimens for analysis

We queried the Tumour Registry database of the University of California, Davis Medical Center for the 4-year period of 1997–2000 and identified 94 cases of adenocarcinoma of the pancreas. These included surgically resected specimens, intraoperative biopsies, and percutaneous biopsies of metastases. Haematoxylin/eosin-stained slides for all specimens were re-reviewed by a single pathologist and 16 cases were subsequently excluded from the analysis because of histology other than pure adenocarcinoma (e.g. adenosquamous), primary tumour location other than pancreas (e.g. ampulla), or insufficient archival tissue for immunohistochemical (IHC) analysis. The remaining 78 tumours of confirmed pancreatic adenocarcinoma serve as the study group and were re-reviewed by the same pathologist to determine the histologic grade (well-differentiated, moderately well differentiated, or poorly differentiated).

### Immunohistochemical technique

From formaldehyde-fixed, paraffin-embedded tumour specimens, fresh representative sections were cut and subsequently rehydrated with xylene and graded alcohols. Antigen retrieval was performed using the microwave technique in 10 mM citrate buffer, pH 6. Both anti-HER-2/*neu* and anti-phospho-AKT antibodies were used at 1 : 50 dilutions. Visualisation was performed using biotinylated horse-antimouse, streptavidin-HRP system (Vector Labs, Inc., Burlingame, CA, USA) followed by 3,3′-diaminobenzidine (Sigma Chemical Co., St Louis, MO, USA). Sections were counterstained with Gill's haematoxylin. Infiltrating ductal carcinoma from a breast cancer specimen was used as a positive control for the HER-2/*neu* IHC, and the LNCaP prostate cancer cell line harbouring a constitutively activated AKT by virtue of a PTEN mutation was used as the positive control for phospho-AKT (pAKT) (Huang *et al*, 2001).

### Scoring of the IHC staining

Scoring of HER-2/*neu* expression used standard quantification as follows: 0 for no staining; 1+, barely perceptible staining not totally encircling the membrane; 2+, light to moderate staining totally encircling the membrane; and 3+, moderate to heavy staining totally encircling the membrane (Yamanaka *et al*, 1993). HER2/*neu* overexpression was classified as 2+ or 3+ with 25% or more cells staining positive (Koeppen *et al*, 2001). Immunohistochemical staining of tumour specimens for pAKT was performed using a previously reported scoring system (Malik *et al*, 2002). In brief, specimens were scored as pAKT positive if >20% or=20% of the tumour cells demonstrated staining at an intensity equal to or greater than that of the positive control. Statistical analysis was performed using *χ*^2^ analysis with significance determined by *P*<0.05.

## RESULTS

### Levels of HER-2/*neu* expression and activation of AKT in pancreatic cancer cell lines *in vitro*

**Figure 1:**
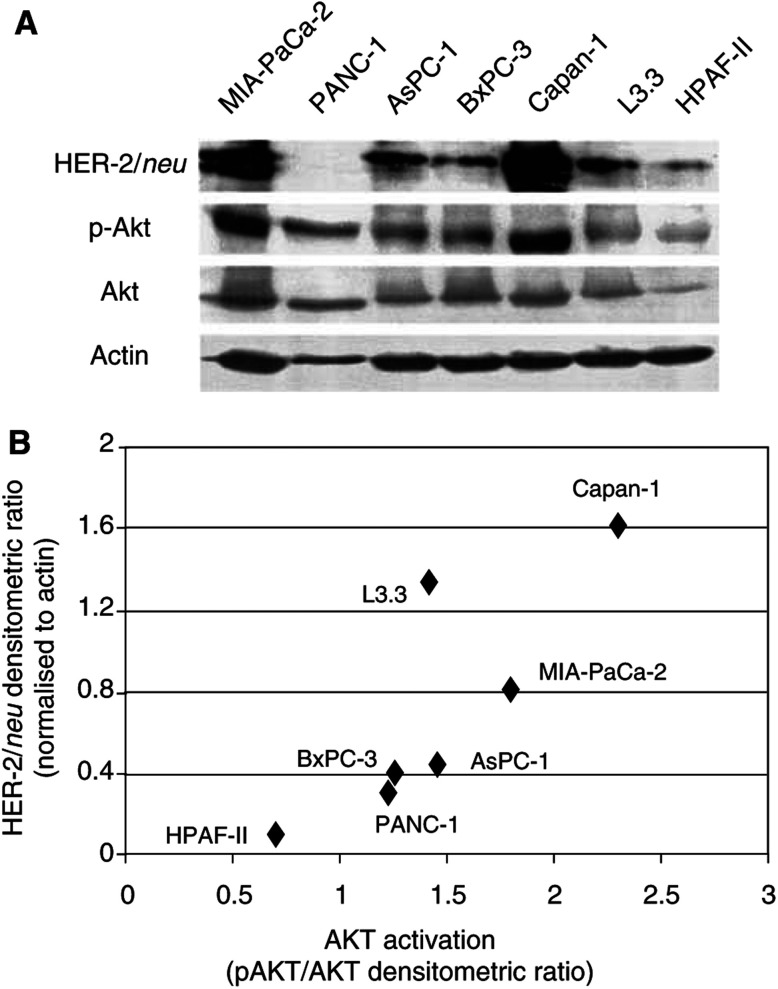
(**A**) Levels of HER-2/*neu*, phospho-AKT (serine-473), and AKT in seven human pancreatic cancer cell lines (MIA-PaCa-2, PANC-1, AsPC-1, BxPC-3, L3.3, CAPAN-1 and HPAF-II). Western blotting for actin was used as a loading control. (**B**) Relationship of level of HER-2/neu expression to activation of AKT (ratio of phospho-AKT to AKT) determined following densitometric quantification of Western blot data (coefficient of correlation=0.82).

We determined the level of HER-2/*neu* and AKT as well as activated AKT (using a phospho-specific antibody) under basal conditions by Western blotting (Figure 1A

). The level of HER-2/*neu* varied among the cell lines with CAPAN-1 and MIA-PaCa-2 demonstrating the highest level. While AKT levels were fairly similar among most of the cell lines, pAKT was highest in the two cell lines (CAPAN-1 and MIA-PaCa-2) with the highest level of HER-2/*neu*. When densitometric quantitation of the level of HER-2/neu expression was correlated with relative degree of activation of AKT (the ratio of the pAKT to AKT densitometric evaluation), a linear relationship was observed for six of the seven cell lines (Figure 1B). pAKT was detectable in all cell lines, even the PANC-1 lacking HER-2/*neu* expression, indicating that upstream signals other than HER-2/*neu* are involved in the constitutive activation of AKT in these pancreatic cancer cell lines.

### Effect of HER-2/*neu* inhibition on pAKT

**Figure 2:**
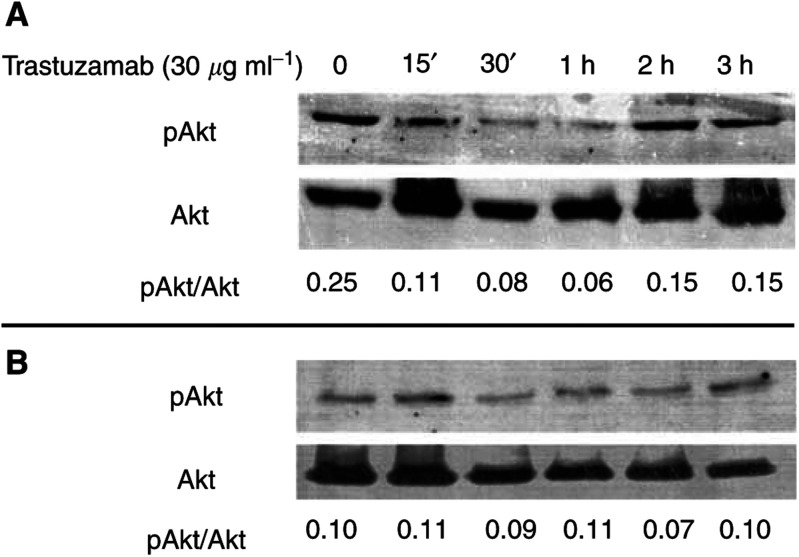
Time-dependent decrease in phospho-AKT following inhibition of HER-2/*neu* with trastuzamab (30 *μ*g ml)^−1^ in MIA-PaCa-2 (**A**) or PANC-1 (**B**) pancreatic cancer cells. Inhibition was observed following 30 min of treatment and returned to baseline after 2 h of treatment. AKT levels are shown for equivalency of loading. Data of densitometric quantification of protein levels are shown below each lane (phospho-AKT normalised to AKT level).

To determine the functional coupling of HER-2/*neu* to AKT activation, the MIA-PaCa-2 and PANC-1 cell lines were treated with the neutralising/blocking monoclonal anti-HER-2/*neu* antibody trastuzamab (Herceptin®). MIA-PaCa-2 cells demonstrated a time-dependent decrease in pAKT, beginning at 30 min and terminating after 2 h of treatment (Figure 2

). This treatment had no effect on total AKT levels, nor any effect on pAKT levels in the PANC-1 cell line that lacks detectable HER-2/*neu* expression.

### Incidence of activated AKT in pancreatic adenocarcinoma tumours

The 78 tumours represented a mix of localised tumours that underwent resection (*n*=35; 45%) and metastatic tumours for which a biopsy was performed for histopathologic diagnosis (*n*=43; 55%). Histologic grading demonstrated that almost one-half of tumours were moderately well differentiated, one-fifth were well differentiated, and one-third were poorly differentiated (Table 1

**Table 1:** Demographics of the patients (*n*=78) whose tumors were examined for AKT activation, HER-2/*neu* overexpression and histologic grade

**Demographics**	***n*=78**
Age (±standard deviation) (years)	65±12
Gender (M : F)	35 : 43
Surgically resected (Y : N)	35 : 43
	
*Histologic grade*	
Well-differentiated	16 (21%)
Moderately well-differentiated	37 (47%)
Poorly differentiated	25 (32%)
	
AKT activated	46 (59%)
HER-2/*neu* overexpressed	52 (67%)

**Figure 3:**
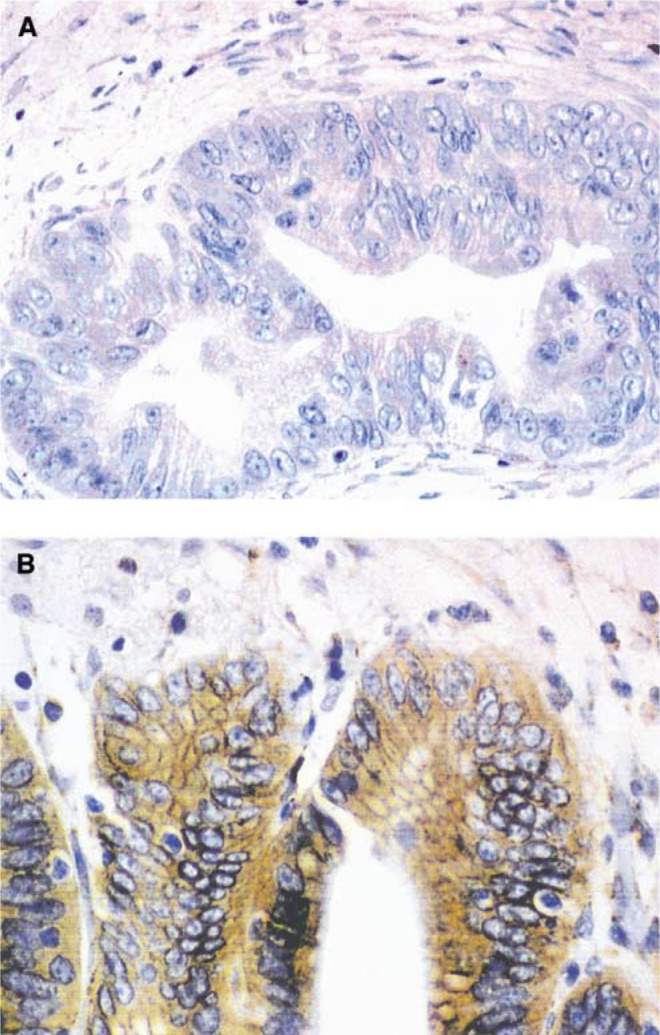
Representative IHC staining for HER-2/*neu* in pancreatic tumours: (**A**) tumour without detectable expression and (**B**) tumour with strong expression including intense membrane staining consistent with overexpression of HER-2/*neu* (× 40, original magnification).

M=male; F=female; Y=yes; N=no.

). Of these 78 tumour specimens, 46 (59%) demonstrated activation of AKT by virtue of IHC staining for pAKT (Figure 3

). The typical pattern of pAKT staining we observed included both cytoplasmic and nuclear immunostaining. This is consistent with most other reports for malignant cells in which the reported subcellular localisation of pAKT staining is cytoplasmic (Gupta *et al*, 2002; Perez-Tenorio and Stal, 2002) and nuclear (Dhawan *et al*, 2002; Itoh *et al*, 2002). Membrane staining is commonly observed in nontransformed cells and has also been reported in malignant cells though usually as a component of cytoplasmic staining (Yuan *et al*, 2000).

### Incidence of Her-2/*neu* overexpression and correlation with pAKT in pancreatic adenocarcinoma

**Figure 4:**
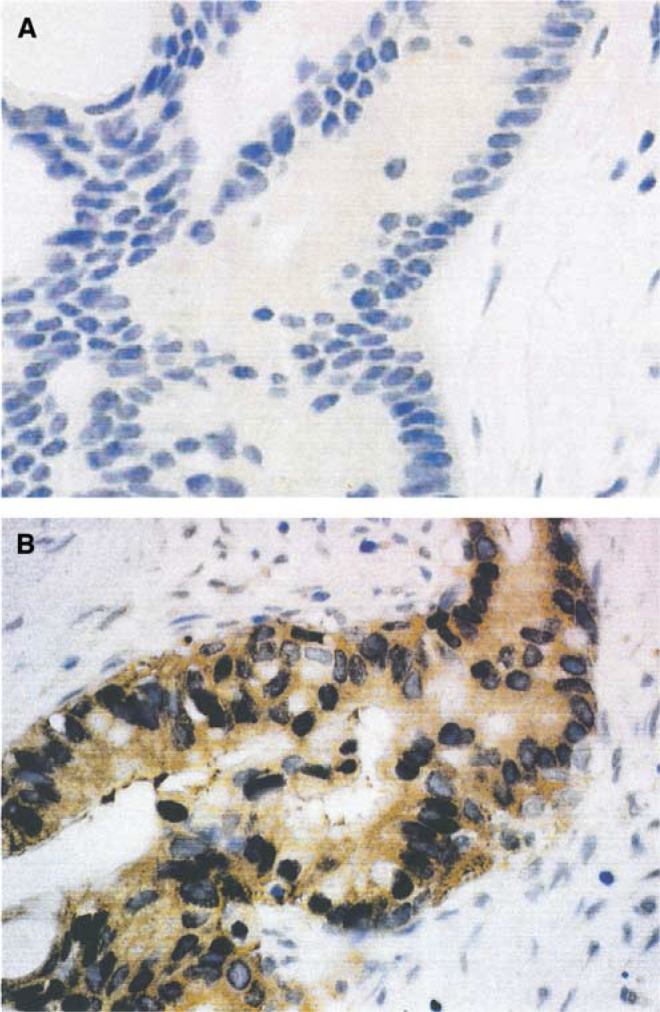
Representative IHC staining for phospho-AKT in pancreatic tumours: (**A**) tumour without detectable expression and (**B**) tumour with strong expression of phospho-AKT consistent with activation of AKT. Normal pancreatic ducts do not have detectable phospho-AKT (× 40, original magnification).

We noted that 51 of the 78 tumours (65%) demonstrated HER-2/*neu* overexpression by immunohistochemistry (Figure 4

). We then correlated HER-2/*neu* overexpression and AKT activation in these 78 pancreatic adenocarcinoma specimens. We found a statistically significant correlation between HER-2/*neu* overexpression and activation of AKT (Table 2

**Table 2:** Correlation between HER-2/*neu* overexpression and AKT activation (*P*=0.015)

	**HER-2/*neu* negative**	**HER-2/*neu* overexpressing**
Phospho-AKT negative	15	17
Phospho-AKT positive	11	35

). Of the 52 HER-2/*neu* overexpressing tumours, 35 (67%) demonstrated increased expression of pAKT; of the 26 tumours lacking HER-2/*neu* expression, 15 (58%) also lacked pAKT expression. These data would indicate that HER-2/*neu* is one of the major upstream activators of AKT; when HER-2/*neu* is not overexpressed, activation of AKT is uncommon, but when HER-2/*neu* becomes an overexpression, the majority of tumours will also harbour activated AKT. Of the 46 tumours that were pAKT positive, 35 (76%) demonstrated overexpression of HER-2/*neu*. AKT can be activated by many upstream receptor tyrosine kinases and this is apparent in the observation that 11 of the 46 (24%) samples demonstrated activated AKT in the absence of HER-2/*neu* overexpression.

### Correlation of activated AKT with histologic grade in pancreatic adenocarcinoma

For patients with pancreatic adenocarcinoma, the single most important prognostic variable is whether the tumour is localised and can be surgically removed (Takahashi *et al*, 1997; Hermanek, 1998; Eskelinen and Haglund, 1999; Kedra *et al*, 2001). Beyond this variable, histologic grade remains one of the most significant prognostic variable, with higher histologic grade (poorly differentiated) being associated with shorter survival. In fact, in studies of mixed populations of localised and metastatic pancreatic cancer, histologic grade is the most significant predictor of survival (Takahashi *et al*, 1997). Survival analysis was not possible in our study population because of two reasons: (1) the patient sample included all stages of pancreatic cancer but with subsets too small for meaningful analysis and (2) survival end points were not consistently available as a significant fraction were referred to UC Davis Medical Center for either the surgical procedure or treatment recommendation, but follow-up was provided by an outside physician. Therefore, we correlated AKT activation with the histologic grade as a surrogate marker of tumour biology. We observed a significant correlation between the presence of activated AKT and higher histologic grade (Table 3

**Table 3:** Correlation between AKT activation and histologic grade (*P*=0.047)

	**Phospho-AKT negative**	**Phospho-AKT positive**
Well differentiated	10	6
Moderately well differentiated	16	21
Poorly differentiated	6	19

). Among the 16 well-differentiated tumours, only six (38%) were pAKT positive, while among the 25 poorly differentiated tumours, 19 (76%) were pAKT positive (*P*=0.047). These data suggest that activated AKT correlates with a marker of aggressive tumour biology in pancreatic cancer.

## DISCUSSION

There has been increasing evidence that AKT is activated in many different cancer types and that this signalling pathway confers a potent survival signal that relates to chemo- and radioresistance (Brognard *et al*, 2001; West *et al*, 2002). Furthermore, early studies have demonstrated that inhibition of AKT sensitises tumours to the apoptotic effect of both chemo- and radiotherapy. The reported incidence of AKT activation varies from 26% in prostate cancer (Malik *et al*, 2002) to 66% in melanoma (Dhawan *et al*, 2002), and aerodigestive squamous cell carcinoma (Gupta *et al*, 2002). Our data demonstrate that AKT is activated in 59% of the pancreatic adenocarcinoma tumours studied, placing it near the top of tumours that have been reported to harbour AKT activation. Furthermore, AKT activation correlated with the histologic grade, which remains one of the most significant prognostic variables in this cancer, surpassing tumour size or nodal status for predicting survival (Takahashi *et al*, 1997; Eskelinen and Haglund, 1999; Luttges *et al*, 2000; Kedra *et al*, 2001).

Although mutation and/or deletion of PTEN can lead to constitutive activation of AKT, it is unknown whether this is the dominant mechanism in tumours other than specific subtypes, such as glioblastome multiforme (Sano *et al*, 1999). Preliminary *in vitro* evidence implicates HER-2/*neu* as a potential mediator of AKT activation with initiation of an antiapoptotic signal (Zhou *et al*, 2001). Furthermore, Zhou *et al* (2000) examined 10 breast cancer specimens overexpressing HER-2/*neu*; seven demonstrated AKT activation. Similarly, only two-thirds of the HER-2/*neu* overexpressing pancreatic tumours demonstrated AKT activation; this suggests that some minor fraction of tumours have an additional defect in the signalling pathway from HER-2/*neu* to AKT that prevents AKT activation despite HER-2/*neu* overexpression. Therefore, if targeted HER-2/*neu* therapy using agents such as trastuzamab is to be employed in pancreatic cancer, and the mechanism of this therapy is mediated by AKT, then it would be anticipated that only tumours demonstrating significant AKT activation would respond. This may explain the mixed results that have been observed in the early clinical trials of trastuzamab in pancreatic cancer (Iannitti *et al*, 2003).

It is also clear that additional upstream mediators other than HER-2/*neu* can activate AKT. In both the examination of the cell lines *in vitro* as well as the tumour specimens, activated AKT was observed in the absence of HER-2/*neu* overexpression. There are numerous genetic events in pancreatic cancer that could potentially lead to activation of AKT, including mutations of either K-ras or PTEN, and constitutive activation of a variety of receptor tyrosine kinases. Therefore, it would appear that HER-2/*neu* overexpression mediates the activation of AKT in some, but not all, pancreatic cancers that demonstrate constitutive activation of AKT. The downstream effectors of AKT that mediate the biologic events are under active investigation. *In vitro* data have coupled AKT to the survival pathway of the transcription factor NF-*κ*B. This is particularly interesting given three observations: (1) the frequent constitutive activation of NF-*κ*B in various cancers including pancreatic tumour specimens (Wang *et al*, 1999), (2) the nuclear immunostaining pattern of pAKT frequently observed in various tumours (Dhawan *et al*, 2002; Itoh *et al*, 2002; Perez-Tenorio and Stal, 2002), and (3) the correlation of pAKT immunostaining and nuclear localisation of the p65 subunit of NF-*κ*B in melanoma specimens (Dhawan *et al*, 2002). We have previously shown in the MIA-PaCa-2 cells that AKT is coupled to NF-*κ*B activation and alters levels of various members of the apoptosis-regulating BCL-2 family (Fahy *et al*, 2003).

Inhibition of both HER-2/*neu* and AKT has been shown to increase the apoptotic sensitivity of pancreatic cancer *in vitro* and *in vivo* (Ng *et al*, 2000, 2001; Buchler *et al*, 2001; Bondar *et al*, 2002; Fahy *et al*, 2003). Interestingly, Buchler *et al* noted that the MIA-PaCa-2 cell line was the most sensitive to the cytotoxic effect of trastuzamab, PANC-1 and AsPC-1 intermediately sensitive and HPAF-II was insensitive. These data closely parallel both the levels of HER-2/*neu* and AKT activation we noted in Figure 1B. While some may promote therapy targeted at AKT independent of mechanism of AKT activation, the *in vitro* data demonstrates the highest degree of AKT activation is observed in those cell lines with the highest level of HER-2/*neu*. Therefore, tumours demonstrating AKT activation by virtue of HER-2/*neu* overexpression may be the most sensitive to therapy directed at AKT. Although this has not been investigated, research into predicting responses to targeted therapy based on molecular and genetic profiles of individual cancers is actively being pursued.

Although AKT amplification has been previously reported in pancreatic cancer specimens (Ruggeri *et al*, 1998), gene amplification does not necessarily correlate with increased kinase activity. Using phospho-specific antibodies, we are now able to determine the activated status of specific kinases and determine whether a specific signalling cascade is functional. Constitutive activation of AKT has been observed in various cancers and represents an evolving target for therapy in cancer. Our data demonstrate that pancreatic cancer may share some of the same biologic characteristics as breast cancer with regards to HER-2/*neu* and AKT signalling, generating some enthusiasm for targeted HER-2/*neu* or AKT therapy in this lethal malignancy.
